# Beyond Just Peptide Antigens: The Complex World of Peptide-Based Cancer Vaccines

**DOI:** 10.3389/fimmu.2021.696791

**Published:** 2021-06-30

**Authors:** Alexander J. Stephens, Nicola A. Burgess-Brown, Shisong Jiang

**Affiliations:** ^1^ Department of Oncology, Medical Sciences Division, University of Oxford, Oxford, United Kingdom; ^2^ Centre for Medicines Discovery, Nuffield Department of Medicine, Medical Sciences Division, University of Oxford, Oxford, United Kingdom

**Keywords:** peptide, cancer, vaccine, dendritic cells, antigen, cross-presentation, immunotherapy

## Abstract

Peptide-based cancer vaccines rely upon the strong activation of the adaptive immune response to elicit its effector function. They have shown to be highly specific and safe, but have yet to prove themselves as an efficacious treatment for cancer in the clinic. This is for a variety of reasons, including tumour heterogeneity, self-tolerance, and immune suppression. Importance has been placed on the overall design of peptide-based cancer vaccines, which have evolved from simple peptide derivatives of a cancer antigen, to complex drugs; incorporating overlapping regions, conjugates, and delivery systems to target and stimulate different components of antigen presenting cells, and to bolster antigen cross-presentation. Peptide-based cancer vaccines are increasingly becoming more personalised to an individual’s tumour antigen repertoire and are often combined with existing cancer treatments. This strategy ultimately aids in combating the shortcomings of a more generalised vaccine strategy and provides a comprehensive treatment, taking into consideration cancer cell variability and its ability to avoid immune interrogation.

## Introduction

### Antigen Presentation in the Context of Cancer

Vaccines have been used for the treatment of infectious diseases for over 200 years and are based on the original principal of inoculating an individual with a weakened or inactive form of a microbe or its constituent components, with the aim of provoking an adaptive immune response to provide long term acquired immunity to a foreign antigen (1). Cancer vaccines work on the same principal by programming the immune system to recognise cancer antigens as ‘foreign’, and can be administered prophylactically to prevent tumour occurrence, or therapeutically as a treatment in individuals who have already contracted the disease.

One of the hallmarks of cancer is its ability to avoid the immune system (2). Normally, aberrant cells are recognised by the immune system through immunosurveillance, where antigen presentation cells recognise and process antigens produced by these cells, presenting them to effector cells and leading to cell death through the adaptive immune response. In cancer however, many factors are at play which prevent the immune system functioning properly. This includes immunoediting, the process by which cancer cells escape the immune system by selective pressure on tumour cells for a non-immunogenic phenotype (3). The tumour microenvironment also plays a part, with its pro-cancer nature promoting tumour growth and preventing a strong anti-tumour response through immunosuppression (4, 5).

The most common treatments for cancers target rapidly dividing cells in a non-discriminatory manner, or by targeting cells with high doses of radiation to damage the DNA of tumour cells and induce cell death. The difficulty however is in the total removal or destruction of cancer cells and inherent or acquired multi-drug resistance, which ultimately leads to tumour recurrence (6, 7). Cancer immunotherapy aims to overcome this by reprogramming the body’s immune system to recognise cancer-specific antigens and the tumours producing them, targeting cancer cells for destruction. This can include the production of anti-tumour antibodies by B-cells through humoral immunity, or through T-cell mediated cytotoxicity through the cell-mediated immune response. For this review, the focus will be primarily on the use of cancer vaccines in the context of a cell-mediated response, and the current progress in the field.

Cancer vaccines require the strong activation of the T-cell mediated adaptive immune response to elicit their anti-tumour potential. The adaptive immune response is initiated by the uptake, processing, and presentation of immunogenic antigens by antigen-presenting cells (APC). Dendritic cells (DCs) are one of the primary professional antigen-presenting cells, and act as the link between the antigen non-specific innate immune response and the antigen-specific adaptive immune response (8). Upon encountering an exogenous antigen, dendritic cells internalise them by receptor-mediated endocytosis or macropinocytosis and process the antigen within endosomes to be loaded onto MHC Class II molecules for presentation to CD4^+^ T cells. This leads to the activation of a Th1 response, including the increased production of cytokines such as IFN-γ, which promotes and maintains macrophages and Cytotoxic T-lymphocyte (CTL) effector functions (9–11). A small proportion of internalised antigen can escape this classical pathway by export into the cytosol of DCs, where they are processed by the proteasome. The resulting peptides are transported to the endoplasmic reticulum, where they are loaded onto MHC class I molecules for presentation to CD8^+^ T cells, a process known as antigen cross-presentation (12). The recognition and subsequent maturation of CD8^+^ T cells by antigen cross-presentation results in an antigen-specific response against cells displaying that antigen. In the case of cancer, the CD8^+^ T cells recognise surface-expressed cancer antigens on tumours and initiate apoptosis through cell-mediated cytotoxicity by releasing apoptotic factors such as Perforin, Fas Ligand and Granzymes (13). The activation of T cells by dendritic cells requires three signals, with any one missing resulting in incomplete activation. The first signal is generated by binding of the T-cell receptor (TCR) to peptide-bound MHC with the aid of the CD4 or CD8 co-receptors, which stabilise the bond and promote TCR signalling (14–16). Signal two is formed from co-stimulatory signals caused by the interaction of cell surface molecules between dendritic cells and T cells, for example by CD28 on T cells with B7 on DCs (17, 18). Finally, the third signal is provided by cytokines released by dendritic cells which drive the T cells into a specific type, for example IL-12 promotes a Th1 phenotype for T-helper cells and promotes the expansion of CD8^+^ T cells (19–21).

Cancer is however, a complicated disease, with immunosuppressive cells in the tumour microenvironment such as regulatory T cells (Treg) and Myeloid-derived suppressor cells (MDSCs) tempering the immune response and aiding in cancer cell immune escape (22). Ultimately, the aim of a cancer vaccine is to strongly activate the CD8^+^ T-cell pathway, mediated by CD4^+^ T cells, thus overcoming self-tolerance and immune suppression, leading to the elimination of cancer cells.

### Principals of Peptide-Based Cancer Vaccines

Peptide-based cancer vaccines typically consist of a sequence of amino acids derived from tumour-specific or tumour-associated antigens (TSA/TAA), the difference being whether the antigen is specific to cancer cells (TSA) or whether it can be found both on healthy and cancer cells, but at elevated levels in cancer (TAA). For peptide-based cancer vaccines to be efficacious, they must contain CD8^+^ epitopes to exploit the antigen cross-presentation pathway, leading to the activation of CTL anti-tumour immunity, along with CD4^+^ epitopes for T-helper cell activation, which sustains CTLs effector function (23). Therefore, to promote a strong immunogenic response, the sequence length of peptide vaccines is important. If the peptide is too short it can bind to MHC of non-professional APCs, which lack the secondary signalling machinery for complete T cell activation, leading to a poor T cell response or immune tolerance (24). Shorter peptides also tend to be HLA-type restricted due to their length not allowing for the diversity required for the high polymorphism of HLA in the general population (25, 26). Finally, short peptides are also prone to enzymatic digestion and elimination from the body faster unless modified (27, 28). A longer peptide length however allows for a broader population coverage of HLA-types (25, 26), the inclusion of multi-epitope peptides to bolster the CD4^+^ and CD8^+^ response, and allows for the inclusion of binding or recognition motifs to bolster immunogenicity.

Peptide-based cancer vaccines have showed promising immunogenicity in a pre-clinical setting, though there is a lot of progress still to be made for them to show strong clinical efficacy – to date no *in vivo* peptide-based cancer vaccine has attained FDA approval (29). There is a multitude of possible reasons for this, including: inappropriate adjuvants (30, 31), tumour heterogeneity (32, 33), tumour antigen loss (34), decreased MHC expression (35, 36), lack of infiltrating T cells in tumour tissue (37), and immune suppression through T cell dysfunction (38, 39).

**Figure 1:**
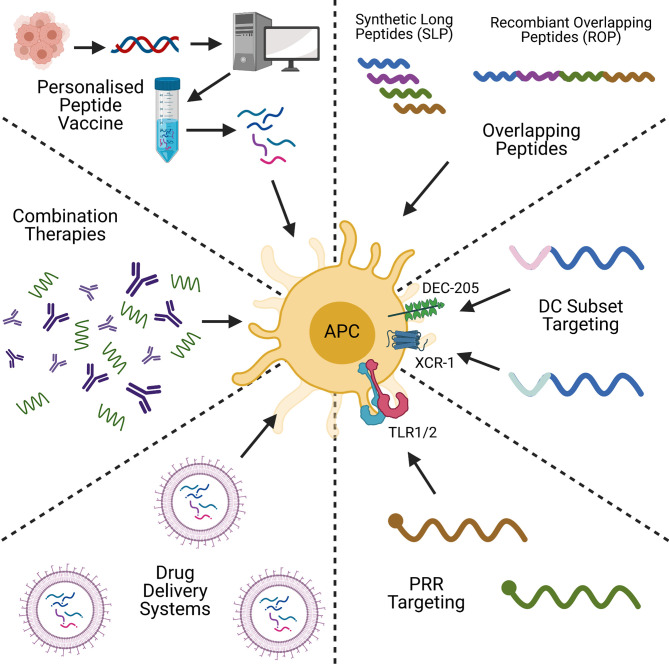
A graphical summary of some of the key concepts in peptide-based cancer vaccine research. Created with BioRender.com.

Peptide-based cancer vaccines stand amongst a plethora of therapeutic strategies for cancer treatment, including DNA/RNA vaccines and adoptive cell transfer (ACT). Like peptide-based cancer vaccines, DNA and RNA-based vaccines are inexpensive to produce, and have the advantage of not being HLA-specific (40). DNA/RNA vaccines are also able to encode multiple antigens that can activate both the adaptive and innate immune responses (41), but DNA vaccines have shown to be poorly immunogenic in humans (42). This is in part due to limited cellular uptake and rapid elimination by the body (43, 44). RNA vaccines are also relatively unstable, and can produce strong unwanted innate immune responses (44, 45), however with modifications to reduce these issues, mRNA vaccines are showing themselves to be promising cancer vaccine candidates (46). ACT on the other hand, functions by taking a patient’s cells, expanding, and engineering them *ex vivo*, before transplanting them back into the body. CAR-T and TIL therapies are examples of this, and have proven to be excellent anti-tumour therapies with a strong and highly personalised immunogenic profile (47). ACT is however an expensive, time- and labour-intensive process (48, 49), and can lead to toxic effects, as seen with cytokine release syndrome in CAR-T patients (50). Often ACT is combined with other cancer vaccine types, including pulsing DCs with tumour antigen-derived peptides, or transfecting with tumour-associated antigen mRNA (51). The hope of peptide-based cancer vaccines is in bridging the gap between these two alternative strategies by being highly specific, with a low manufacturing cost, and a proven safety record (52). However, challenges remain in improving their immunogenicity and attaining use in the clinic. The aim of this review is to evaluate recent strategies to improve the immunogenicity of peptide-based cancer vaccines, and to look for trends which could lead to their clinical application. The topics of discussion will be on peptide design, conjugation, formulation, personalised peptide vaccines, and combination therapies (**Figure 1**). We will discuss how each strategy overcomes the issues highlighted and the future of peptide-based cancer vaccines.

## Designing Peptide-Based Cancer Vaccines

### Long Peptides and Overlapping Peptides as Cancer Vaccines

Peptide-based cancer vaccines are established on the principle of selecting peptide sequences from TSA/TAAs containing T cell epitopes to use as a template. This can take the form of single epitopes, long-chained polypeptides with multiple epitopes, or pools of peptides. However, to produce a strong immunogenic response, peptide-based cancer vaccines need to include multiple epitopes that are recognised by both CD8^+^ and CD4^+^ T cells in a diverse population with different HLA haplotypes. The rationale of this strategy being that, unlike the primarily structural-based recognition of antibodies (53), T-cell receptors recognise short linear peptide sequences derived from an antigen. It is therefore possible to use *in silico* bioinformatics and T cell epitope mapping to predict and select sequences from a target tumour antigen (53–55). By using multi-epitope or overlapping peptide sequences as opposed to short single-epitope peptides, issues such as tumour heterogeneity, tumour antigen down-regulation and the diversity of HLA haplotypes may be overcome.

One type of peptide-based cancer vaccines is synthetic long peptides (SLPs), which are pools of 25-35 amino acid peptides derived from TAA/TSAs. SLPs have successfully shown to elicit a strong immunogenic response since their inception (56–58), and have proven to be more immunogenic compared to the whole antigen in which they are derived from (59). Using long peptides as opposed to short peptides equal in size to T cell epitopes, means that the peptide must be processed within dendritic cells before they can be presented to T cells, rather than binding directly to MHC-I of dendritic cells or non-APCs (60, 61). However, a pool of peptides will need to be quality controlled for each individual peptide within that pool which could hamper its manufacturing capability and cost.

Recently SLPs derived from MELOE-1 melanoma antigen have been developed from Class I and Class II epitopes separated by a cathepsin protease-sensitive linker (62). Cathepsins are key proteases in dendritic cells involved in antigen presentation (63, 64), and it was found that the composition and size of the Cathepsin-sensitive linker had a significant impact on the presentation of the CD4^+^ and CD8^+^ T cell epitopes. Of the linker sequences tested, LLSVGG showed the strongest immunogenicity (62). Mouse studies to evaluate SLPs in the prime-boost immunisation strategy using LLSVGG-based vaccine showed a strong CD8^+^ T cell response, but a lower CD4^+^ compared to human PBMC tests, which could produce a less well-rounded response, and shows the epitope sensitivity differences between mice and human models (62). Mouse tumour models also revealed a reduction in tumour growth in 4 out of 7 mice when compared to unvaccinated mice but fell outside of significance (62). To help clarify these results, further studies with an alternative antigen model that shows similar epitope reactivity between species, as well as an increased sample size are needed. This study demonstrates the flexibility of SLP technology in peptide vaccine design, through the incorporation of strategic and specific enzymatic cleavage sites to enhance antigen presentation.

Among many others, Survivin is a cancer antigen that has been the target of peptide-based cancer vaccine design. Survivin is an anti-apoptotic protein and a member of the inhibitor of apoptosis family. It is a classic tumour-associated antigen that is not normally found in somatic cells but is up-regulated in numerous cancers (65). A Survivin-based vaccine composed of a pool of three SLPs with eight CD4^+^ epitopes and six CD8^+^ epitopes was recently developed (66). Although Survivin is found in a large proportion of cancer cells, it is normally immune tolerant (67, 68), the Survivin-SLP vaccine however was shown to activate both CD4^+^ and CD8^+^ immune responses through stimulation with autologous dendritic cells, regardless of HLA types in tested populations (66). Following on from this, the SLP vaccine in engrafted mouse models for colorectal cancer and B-lymphoma showed a significant level of tumour eradication, with secondary challenge also demonstrating reduced tumour growth and complete survival up to 60 days (66). The cytokine release profile of CD4^+^ and CD8^+^ T cells were enhanced with the vaccine, and with an increase in Perforin and Granzyme B, which forms a part of the CTL response (13, 66).

Recombinant overlapping peptides (ROPs) are another design strategy for peptide vaccines which have shown promising pre-clinical efficacy. ROPs are comprised of sequential overlapping long peptide sequences covering the whole sequence of a target, with Cathepsin S protease-sensitive linkers between the peptide sequence overlaps (69, 70). The overlapping region allows for diversity in epitope, in particular with MHC-II molecules which have shown different but overlapping recognition of CD4^+^ epitopes between HLA haplotypes (71). ROPs differ from other synthetic peptide pools as they are produced recombinantly as a single-chain polypeptide with multiple epitopes, giving ROPs potential advantages in manufacturing and drug approval. However, dealing with long peptides also comes with problems in solubility. ROPs have shown to produce strong immunogenic response in both CD4^+^ and CD8^+^ T cell over native protein, and are able to break self-tolerance as shown with Survivin ROP, mainly due to its design resulting in reduced homology when compared to native protein (70).

Overcoming immune tolerance is a significant hurdle for peptide-based cancer vaccines, as T cells reactive to self-antigens are eliminated as part of the central and peripheral tolerance mechanisms. But by incorporating multiple epitopes for CD4^+^ and CD8^+^ T cell activation, the examples so far have shown to be immunogenic in diverse HLA types which may not be possible with single epitope vaccines.

### Personalised Peptide-Based Cancer Vaccines

Peptide vaccine design is key in targeting tumour neoantigens, and with the emergence of whole exome sequencing (WES) and single-cell RNA-Sequencing (RNA-Seq), peptide-based cancer vaccines are increasingly becoming more tailored to an individual’s neoantigen repertoire. By creating specific vaccines to each patient and their genetic background, personalised immunotherapy avoids the issues of “off-the-shelf” peptide vaccines which may not take into consideration tumour heterogeneity and HLA haplotype. Reports in this field are promising, for example, by combining WES and RNA-Seq with *in silico* neoepitope predictions, Ott P.A. et al., created 20 unique SLPs specific to patient HLA type (72). *Ex vivo* experiments showed a strong CD4^+^ antigen specific response while CD8^+^ response was undetectable until after a round of *in vitro* expansion with the peptides (72). Indeed, the weaker CD8^+^ T cell response might be explained by only 16% of the peptides containing CD8^+^ T cell epitopes compared to 60% for CD4^+^ epitopes (72). This could show a bias in the software for predicting of CD4^+^ epitope sites or the higher level of promiscuity of MHC-II peptide binding compared to MHC-I (73). Further experiments showed that the CD4^+^ and CD8^+^ immune responses were highly specific, with 86% of T cells acting against the mutant antigens but not the wildtype (72). In a phase I vaccination trial, four stage IIB/C patients were disease free after 2 years, with another two patients in Stage IVM1b requiring further treatment with anti-PD-1 therapy to achieve tumour regression (72). Another example is in the clinical study by Hilf N. et al., that looked at personalised vaccination strategies against glioblastoma (74). Glioblastoma is notorious for its bad patient prognosis, with mean survival with Temozolomide and radiotherapy of 14.6 months (75). In the phase I study, a two-pronged vaccine treatment strategy was adopted using a pre-made library of unmutated antigens from glioblastomas and ranking each patient’s response to them. This was followed with a second vaccine consisting of either; mutated antigen peptides predicted to bind MHC-I and produce an immune response, or any other unmutated epitopes not included in the first vaccine’s library (74). For the first vaccine, around half of the total evaluated peptides were CD8^+^ and CD4^+^ immunogenic, with the CD8^+^ showing a primarily memory phenotype, while the second vaccine was dominated by a Th1 CD4^+^ response (74). It is worth considering that the original idea of the second vaccine was to use next-generation sequencing to compare the patient’s genomic mutations against HLA-bound peptides by mass spectrometry, but that failed to match any (74). What this research demonstrates is the infancy of personalisation strategies, but also showing its promise as a highly specific treatment for individuals. The main issues with neoantigen-based peptide vaccines at present are the cost and time required to produce, but advances in sequencing, software predictions, databasing and manufacturing hope to allow for their use on a larger, more cost-effective scale (76).

## Peptide-Conjugate Vaccines

### Targeting Pattern Recognition Receptors

Peptide-based cancer vaccines alone are poorly immunogenic and require strong adjuvants or immune stimulants to produce an efficacious response. But by combining peptide-based cancer vaccines with conjugates that stimulate or target dendritic cells, peptide-conjugate vaccines have shown a greater potential over peptide vaccines alone. One common method is the inclusion of dendritic cell activation markers such as pathogen associated molecular patterns (PAMPs), or damage associated molecular patterns (DAMPs), to act as adjuvants by binding to pattern recognition receptors (PRRs) on the surface of dendritic cells. Examples of PRRs include the toll-like receptors (TLR), C-type lectin receptors (CLR) and NOD-like receptors (NLR). The activation of PRRs induces the maturation of DCs, causing an upregulation of MHC-II expression, co-stimulatory signalling, and the release of pro-inflammatory cytokines, to bolster the anti-tumour response (77–80). PRRs in the context of peptide-based cancer vaccines varies from a simple adjuvant mixed with the peptide vaccine (81), to PRR ligand-peptide conjugates.

One example of this novel technology is a conjugate formed from the TLR2 ligand Pam3CSK4 covalently bound to TLR1 (82). As TLR1 is TLR2’s heterodimeric partner, the conjugate enhances the targeting of TLR1 to TLR2 for dimerization and by proxy the immunogenicity of the SLP it is conjugated to (82). Research from this group showed a significant increase in the frequency of SIINFEKL (an OVA CD8^+^ epitope) positive H-2kb^+^ cells with the use of SLP-Conjugate over Pam3CSK4 alone, with significantly more DC maturation markers (83). They also showed greater CD8^+^ T cell infiltration in a HPV16 TC-1 tumour model, with a reduction in tumour growth and increased survival time (83, 84).

### Targeting Dendritic Cell Subsets

A further method of targeting dendritic cells is by incorporating ligands specific to DCs or a subtype of DCs (85). DEC-205 (CD205) is a dendritic cell receptor involved in receptor-mediated endocytosis, and has been associated with antigen cross-presentation in CD8^+^ dendritic cells (86). Although its natural ligand is not fully understood, there is some evidence of its involvement as a PRR in binding to CpG oligodeoxynucleotides and recognition of apoptotic cells (86, 87). In theory, by conjugating a cancer peptide to a ligand recognised by DEC-205, the vaccines antigen presentation ability could be enhanced. Recently, Liu Z. et al., designed a ScFv that targets the DC marker DEC-205 fused with a HPV E7 SLP, which showed potent targeting effect when compared with the SLP alone (88). However, the author notes that the conjugating motif used in the study stimulates a CD4^+^ response by itself (88). While this is not necessarily a negative, may have exaggerated the DEC-205 ScFv potency to target DCs.

Along with the PRRs already mentioned, DCs contain many chemokine receptors which are used in their migration or in attraction of other cells of the adaptive immune response. One example is the XCR1 receptor, a chemokine receptor which binds XCL1 to attract DCs to CTLs (89). What is of particular interest about XCR1^+^ DCs, is their high efficiency at antigen cross-presentation (90). Botelho N.K. et al., investigated XCL1 fused with OVA SLP and a mutated IgG1 Fc which prevents Fc-mediated endocytosis, to evaluate its immunogenic potential in OVA expressing tumour models (91). The inclusion of the XCL1-Fc fused to the OVA peptide showed significant anti-tumour immunity in B16-OVA tumour models, with increased CTL response when compared to OVA SLP alone and OVA with free XCL1 (91). Interestingly the inclusion of OVA SLP with free XCL-Fc showed very similar responses than the full fusion, the author speculates this may be caused by aggregation of the molecules (91). It is worth noting this paper did not consider the equally important CD4^+^ response, which would provide valuable insight into the viability of this targeted strategy as an anti-cancer treatment.

### Multiple-Conjugate Peptide-Based Cancer Vaccines

So far, all the conjugates given as examples have focused on one target or conjugate per peptide vaccine. Logically, by combining multiple conjugates with different effector or targeting motifs, peptide-conjugate vaccines can synergistically boost multiple branches of the adaptive immune response. One example of this was recently developed by a combination of a cell-penetrating peptide (CPP), a TLR2/4 agonist which activates APCs, and a multi-antigenic domain that stimulates CD4^+^ and CD8^+^ T cells (92). The TLR agonist promotes DC maturation and activation, while the cell-penetrating peptide allows the antigenic domain to access the cytosolic compartment of DCs where antigen cross-presentation occurs, increasing production of antigen-specific CD8^+^ T cells and boosting anti-tumour immunity (93). With this strategy, in HPV TC-1 therapeutic mouse tumour models, a significant increase in survival time and reduction in tumour size were observed, and in multiple mouse models an increase in antigen-specific CD8^+^ T cell tumour infiltration (92). Finally, they found in non-human primates the ability to break self-tolerance by eliciting a T cell response to EPCAM and Survivin (92). It is worth considering though that CPPs are non-specific and will penetrate most cells (94), possibly leading to substantial off-target effects and reduced bioavailability to DCs through absorption by non-professional APCs. Perhaps a method of combing the ability of CPPs to penetrate cells in a more targeted approach to DCs could be beneficial to the creation of an efficacious peptide-based cancer vaccine.

## Peptide Vaccine Formulation & Drug Delivery Systems

### PLGA and Liposomes as Particulate Drug Delivery Platforms

The shortcomings of peptide-based cancer vaccines can be improved by proper formulation. For example, incorporating drug delivery systems into the vaccine formulation can facilitate peptide delivery to antigen presenting cells. By using drug delivery systems, peptides along with adjuvants and targeting motifs, can be encapsulated, or incorporated onto a surface, allowing for delivery of a single “package” to protect the peptide and deliver a strong T-cell mediated response.

PLGA and liposomes are two examples of drug delivery systems which have been used experimentally for many years, and have a proven track-record in safety and biodegradability, with the FDA approving their use as drug delivery systems (29, 95). Liposomes are highly customizable cell membrane mimics composed of phospholipid bilayers. The charge, size, surface molecules and delivery mechanism of liposomes are all tailorable – this feature allows a liposome to mimic the size and surface markers of a pathogen for example (96, 97). As particulate systems can protect peptides from degradation and control their release, liposomes provide peptides greater access to the spleen and lymph nodes which contain a higher proportion of cross-presenting DCs (98, 99). Upon internalisation, the liposome can continue to promote antigen cross-presentation by enabling its peptide cargo to escape from the lysosome into the cytosol, a key step in antigen cross-presentation and stimulation of a robust CD8^+^ T cell response (100).

One example of the diversity of liposome-based delivery systems is in nanoliposomes designed by Rueda et. al., which contained multi-antigenic T-helper cell epitopes against LHR hormone, tetanus toxin immunogen as an adjuvant, and external Fc receptor ligands which increases liposome uptake by DCs (101). The adjuvants bolstered DC maturation, and the inclusion of multiple independent TLR agonists worked synergistically to enhance the stimulation of DCs *in vitro* (101). However, the efficacy of this strategy with tumour models both *in vitro* and *in vivo* was not investigated, which is needed to fully evaluate its anti-tumour response against the self-antigen LHRH for the treatment of prostate cancer.

In another example of the customisability of liposomes, and how the lipid composition can affect antigen uptake, Zamani, P. et al., designed a DOPE-liposome system in combination with monophosphoryl lipid A (MPL), a detoxified LPS adjuvant derivative, and Pan HLA-DR epitope (PADRE) peptide (102). PADRE is considered a ‘universal’ HLA-DR (MHC class II) restrictive CD4^+^ epitope, which stimulates a CD4^+^ response in most patients (103). By using DOPE in the liposome design, the nanoparticle forms a hexagonal structure at low pH, which permits the particle to fuse with the endosomal wall and escape into the cytosolic pathway for MHC Class I cross-presentation (102, 104). The authors combined the DOPE : PADRE : MPL liposome with P5 peptide derived from HER2/neu breast cancer epitopes, and found reduced tumour growth and increased survival time in mice vaccinated with the P5^+^DOPE : PADRE : MPL liposomes (102). A second study using a different HER2/neu derived peptide, showed similar results with an increased presence of CD4^+^ tumour-infiltrating lymphocytes as well (105). Together these studies show that by optimising vaccine formulation, it is possible to re-direct antigen presentation pathway from MHC-I to MHC-II. However, optimisation is important and necessary as the weak CD4^+^ cytokine profile and no apparent reduction in Treg cell numbers within the TME (102), may harm the vaccine’s efficacy in the clinic.

In a study that looked at mutant KRAS SLP-Liposomes, the use of KRAS G12 mutant SLPs alone resulted in primarily a CD4^+^ response (99). It was only upon the SLP being bound to the liposome did the vaccine produce a strong CD8^+^ response, albeit at the slight expense of CD4^+^ activity (99). The authors did note an increase in tumour PD-1 and TIL exhaustion markers, which resulted in a therapeutic response that slowed tumour growth but did not lead to regression (99). However, upon combination with PD-1 checkpoint inhibition therapy they saw tumour regression in 5 out of 10 mice with the Neo-lpx vaccine (99). One highlight of the work was the remarkable specificity of the vaccine to the mutant KRAS and not to the wildtype (99), emphasising the safety of formulated peptide-based cancer vaccines.

The importance of formulating an appropriate particle-based delivery system is imperative to the efficacy of a peptide-based cancer vaccine. This was proven by comparing PLGA and liposome with free peptide (106). It was found that although using a particulate based system was better than free peptide with adjuvant, liposomes were consistently better than PLGA at eliciting an anti-tumour immune response (106). The possible reason being the cationic charge of the liposome, and its comparatively smaller size than PLGA, promoting a stronger attraction and elevated uptake of the liposome by DCs (106).

One caveat for formulating vaccine delivery systems is the complications of construction and manufacture. Jacoberger-Foissac, C. et al., demonstrated this by looking to optimise liposome delivery by experimenting with different CD4^+^ and CD8^+^ epitopes in combination with adjuvants (107). By experimentation and sequential screening, they displayed the versatility and modular nature of liposomes as a delivery system. However, they also highlighted the empirical nature of its construction and the difficulties in manufacturing and optimisation.

### Novel Delivery Systems for Peptide-Based Cancer Vaccines

So far, the focus has been on the use of PLGA and liposomes, but many groups are exploring novel formulations for peptide-based cancer vaccine delivery systems and their composition. For example, it has been shown that simply using the amino acid L-Tyrosine in combination with an adjuvant formula acts as a depot for peptide vaccines. This effect could enhance the duration and effectiveness of the peptides, and was found to work similarly to repeated injection of peptide alone (108). Although ultimately the study showed the strategy to be no better than repeated vaccination, this depot effect still has its benefit in allowing fewer vaccinations to attain the same effect.

Cross-linked polymer networks known as nanogels are also being explored, which can be customised with different sizes, charges and properties that allow the release of their payload by a trigger such as pH or enzymatic cleavage (109–111). Indeed, one group took advantage of this by designing a nanogel that releases its peptide payload in a reducing environment, as is found in endosomes (109). They found in *in vitro* and *in vivo* experiments that the nanogel vaccine was superior to soluble SLP in stimulating CD4^+^ and CD8^+^ response with adjuvant, although the CD4^+^ response was not as strong *in vivo* than *in vitro* (109).

Finally, one group designed an ingenious delivery platform for their PPV consisting of a charge modified TLR7/8a conjugate, that was able to self-assemble into precise 20 nm diameter particles regardless of peptides it was conjugated to (112). The self-assembled particles were able to induce a CD8^+^ T cell response 20-fold higher than PLGA and liposomes with the same dose (112). *In vivo* experiments also showed a larger accumulation of nanoparticles in the lymph nodes compared with soluble SLP and microparticles (particles greater than 200 nm in diameter), as well as producing a higher CD8^+^ T cell response and a significant reduction in tumour growth rate in M39 mice (112). As this system seems to work regardless of the peptide load, it could reduce the empirical testing required by traditional carrier systems, while simultaneously reducing the variability of peptide loading and potential damage to peptide integrity.

## Peptide-Based Cancer Vaccine Combination Therapies

### Peptide-Based Cancer Vaccines and Immune Checkpoint Inhibitors

Peptide-based cancer vaccines as a monotherapy have yet to show an efficacious response in the clinic. However, data to date has shown that peptide-based cancer vaccines can work in combination with other drugs or therapies to enhance efficacy over individual monotherapies. One prominent example is the combination of peptide-based cancer vaccines with checkpoint inhibitors, such as anti-PD-1. Checkpoint blockades act as the brakes of the immune system to regulate T cell response, and are essential for self-tolerance and prevention of autoimmune disorders. However, the checkpoint blockade also scuppers cancer immunotherapy by supressing effector CTL function on tumours (113, 114). Checkpoint inhibitors block this action, overcoming immune suppression and allowing for greater antigen-specific T cell responses against tumours. By combining checkpoint inhibitors with peptide-based cancer vaccines, the immune system is released from suppression, allowing it to specifically target cancer cells.

Many of groups in this review combined their therapy with checkpoint inhibitors and additional anti-cancer agents to test efficacy in combination therapies. Liu, Z. et al., found their DEC-205- targeting ScFv-HPV E7 SLP fusion resulted in higher PD-L1 expression, and were able to show a more efficacious response when combining anti-PD-L1 antibody with their vaccine (88). Zom, G.G. et al., had a curative rate of 10% with their Pam3CSK4-TLR-SLP fusion as a monotherapy, however when used in combination with the cervical cancer chemotherapy drug Cisplatin survival increased to 71%, and with photodynamic therapy survival increased to 89% (83). The authors cited the possible reasons to be; depletion of immunosuppressive myeloid cells, increased TNF-α sensitivity, or induction of immunogenic cell death (83). Finally, Belnouse, E. et al., found that combining their modular self-adjuvating vaccine strategy, composed of a CPP with a multi-antigenic domain and a TLR2/4 agonist, with anti-PDL1 therapy, achieved greater efficacy than the vaccine alone (92). This perfectly illustrates that even with targeting motifs or PRR agonists to enhance peptide vaccine immunogenicity, peptide-based cancer vaccines as a monotherapy are still inferior to combination with other treatment strategies. This being attributed to the complexity of tumour immunology and the suppressive nature of the tumour microenvironment.

### Combining Peptide-Based Cancer Vaccines With Existing Cancer Therapies

Combining peptide-based cancer vaccines with existing anti-cancer therapies is common, as patients are often treated with established chemotherapy, radiotherapy, and immunotherapy as part of standard care practices. Trastuzumab for example is an anti-HER2 monoclonal antibody used to treat breast cancer, and has been shown to make HER2^+^ tumour cells more susceptible to antibody dependent and T-cell mediated cytotoxicity (115, 116). In one study, anti-HER2 antibodies enabled DCs to expand HER2-derived peptide E75 specific CTLs greater than peptide alone (117). *In vivo* experiments with anti-HER2 antibodies showed similar increases in antigen-specific CTLs with spontaneous and implantable HER2 mouse models (117). In a phase IIb clinical trial combining Trastuzumab with GM-CSF and E75, the vaccine was found to be safe and non-toxic (118).

Another group combined their peptide pool with docetaxel, a standard of care chemotherapy drug for treatment of castration-resistant prostate cancer (119). Docetaxel has been shown to reduce immunosuppression within tumours by reducing Treg cell numbers (120), and it was thought that combining Docetaxel with a peptide vaccine may enhance its efficacy (119). Unfortunately, in a randomised phase II trial, the combination did not show a robust synergistic effect, with no increase in overall survival, even with a decrease in PSA levels and a reduction in the immunosuppressive MDSCs (119).

Cyclophosphamide (CPA) is a chemotherapy agent with direct cytotoxicity in high doses, but has immunomodulatory effects when used at lower doses, including the suppression of Treg cells and the modulation of antigen-specific T cell responses (121). In a randomized phase II trial that investigated the efficacy of combining personalised peptide vaccines (PPVs) with CPA on previously treated advanced biliary tract cancer patients (122), pre-vaccinated PBMCs showed no significant increase in IFN-γ with the use of PPV compared with PPV and CPA combination therapy (122). However, in the clinical context they saw a doubling of progression free and overall survival and a reduction in IL-6 with the PPV/CPA combination compared to PPV alone; lower IL-6 is suggested to be associated with a better prognosis (123, 124). However, the expected Treg reduction shown to occur with low dose CPA treatment did not occur when using PPV/CPA combination, and a mixed picture was observed with frequency and numbers of MDSCs, which did not correlate with an increase in overall survival (121, 122). Taken together, this suggests that although the results related to clinical outcomes were promising, more research is necessary to optimise the combination of PPVs with CPA.

The mixed results between the three studies highlighted, emphasises the need for careful consideration on the design of experiment and the need for empirical investigation into the combinations worth pursuing. Especially when working with combination therapies where changing variables such as dose, administrative route and timing can have drastic implications on a vaccine’s capability.

### Novel Therapies That Modulate Peptide-Based Cancer Vaccine Function

Some of the drugs being investigated in combination with peptide-based cancer vaccines do not have direct anti-cancer properties, but help to modulate mechanisms required for peptide-based cancer vaccines to function. Avasimibe for example is an ACAT1 inhibitor, which prevents esterification of cholesterol and the attenuation of lipid rafts, which in turn increases the level of cholesterol in CD8^+^ T cells and promotes T cell receptor signal transduction, enhancing anti-tumour response (125). One group found that by combining Avasimibe with a KRAS multi-peptide vaccine in prophylactic mouse models, a significant decrease in tumour volume was seen compared to monotherapies, with an increase in CD8^+^ T cell levels in the TME (126). In therapeutic models, the Avasimibe/KRAS combination therapy did not show a significant decrease in tumourigenesis, but did show a reduction in tumour load and delayed tumour progression (126). This again highlights the difference in efficacy that can occur depending on the vaccine setting.

Most of this review has focused on how CD8^+^ T cell activity is imperative to a strong anti-tumour response, with CD4^+^ T cells playing a supporting role in activating and maintaining the immune response. However, there is evidence of the importance of CD4^+^ activity in generating an anti-tumour response directly with so called cytotoxic CD4^+^ T cells. Cytotoxic CD4^+^ T cells are characterised by their ability to produce Granzyme B and Perforin (127, 128). Kumai T. et al., focused on inducing an anti-tumour CD4^+^ response as opposed to CD8^+^, by combining CD4^+^ epitope specific peptides with TLR ligands, CD40 monoclonal antibodies and with various co-stimulatory activators to optimise the CD4^+^ activity (129). OX40 (CD134) was one example of a co-stimulatory activator, which is used to maintain long-term T cell activity by promoting survival and proliferation (130). OX40 agonistic monoclonal antibodies in combination with the CD4^+^ epitope peptide vaccine showed an enhanced peptide-specific CD4^+^ T cell response, and a slowing of tumour progression in therapeutic models, with an increase in IFN-γ, TNF-ɑ and Granzyme B production (129). Interestingly, this would point to a possible cytotoxic CD4^+^ activity, as in CD8^+^ depleted mice there was still a reduced anti-tumour response (129). This study emphasises the importance of considering both the CD4^+^ and CD8^+^ activity when designing a peptide vaccine strategy; it would be interesting to see the results of combining this with a CD8^+^ specific vaccine to observe its effects.

Oncolytic viruses are an emerging therapy that utilise engineered viruses to target and kill cancer cells, with the first oncolytic virus approved by the FDA in 2015 (131). One group combined a Maraba virus engineered to produce E6 and E7 sequences from HPV16 with SLP peptide derived from epitope mapping of HPV16/18 E6 and E7 wildtype sequences (132). By using the SLP as the ‘Prime’ in a prime-boost vaccine strategy, they showed an increase in IFN-γ and TNF-α release by CD8^+^ T cells, but no significant increase in survival time in mouse models compared to SLP prime-boost monotherapy (132). This strategy warrants further investigation into optimising the vaccine and administration strategy, with more trials and differing the peptide target and virus.

## Concluding Remarks

Peptide-based cancer vaccines are a diverse and versatile means of eliciting a cell-mediated anti-tumour response through antigen presentation of tumour antigen epitopes to T cells. The activated T cells then recognise and respond to tumour antigens presented on the surface of cancer cells, initiating an immune response, and subsequently leading to T cell mediated killing of the cancer cells. Many conjugates and polymers are used to enhance the immunogenicity of peptide-based cancer vaccines by targeting the peptides to specific subtypes of immune cells, or by containing stimulatory molecules to increase the activation and maturation of dendritic cells. Many groups have shown promising results combining peptide vaccines with chemotherapy agents, along with drugs not originally designed as anti-cancer agents. Others are incorporating peptide vaccines into highly customisable vaccine carrier systems, bringing together CD4^+^ and CD8^+^ epitopes, adjuvants, and targeting motifs into a single particle. In general, peptide-based cancer vaccines as a monotherapy struggle to achieve efficacy, but show great promise as a component of a combinational treatment strategy. Combination therapy is likely to be the approach needed for peptide-based cancer vaccines to gain traction as a viable treatment in the clinic.

As vaccines increasingly become more customised to individual patients, personalised peptide vaccines represent a promising vaccine candidate. The design and manufacture of personalised peptide vaccines are currently an expensive and time-consuming process, but will be a valuable toolkit in the future with the advent of new sequencing technologies, bioinformatics, T cell epitope prediction and improved manufacturing practices. From this review, one can appreciate the complexity involved with designing a peptide-based cancer vaccine and the challenges of striking a fine balance between method and mode of delivery, half-life, epitope selection, and immunogenicity to produce an efficacious vaccine strategy. Although many of the studies outlined in this review were pre-clinical or in the early stages of clinical trials, studies on peptide-based cancer vaccines in the clinic are numerous. As of May 2021, there are approaching 80 phase I or II clinical trials utilising a peptide-based vaccine strategy in cancer, with 20 currently active and 20 having been completed since the start of 2019 (133). **Table 1** summarises the current Phase I & II peptide-based cancer vaccine trials currently active and/or recruiting. Featuring prominently on the list are peptide-based cancer vaccines against breast, lung, blood and brain cancers to name a few (133). Exhibiting the diversity of targets peptide-based cancer vaccines are being trialled upon (133). What is quite apparent though is the lack of trials beyond phase II, illustrating the current issues with efficacy that peptide-based cancer vaccines face. However, encouragingly there is a clear trend towards a more personalised approach to patient neoepitope selection in the current pool of trials, with an increased focus on peptide-based cancer vaccines use in combination with other cancer treatment strategies (133). For a more detailed analysis, Bezu, L. et al., have expertly collated and reviewed trials up until 2018 for peptide-based cancer vaccines (134).

**Table 1:** A summary of Phase I and II peptide-based cancer vaccine clinical trials currently active or recruiting.

Condition	Peptide Vaccine Type	NCT number
Adenocarcinoma	HER2/neu Peptides	NCT02795988
	Personalised Peptide Vaccine	NCT04627246, NCT02600949
Bladder Cancer	Personalised Peptide Vaccine	NCT03359239
Blood Cancer & Leukaemia	Multiple Peptides	NCT04051307
	Personalised Peptide Vaccine Combination Therapy	NCT04688385, NCT03559413, NCT02802943
	WT1 Peptides	NCT04747002, NCT03761914
	IDO Peptides	NCT03939234
	Survivin Peptides	NCT02334865
Brain Cancer	TAA Peptide Combination Therapy	NCT01795313
	IDH1 Peptide	NCT02193347
Breast Cancer	Folate Receptor Peptide Combination Therapy	NCT02593227, NCT03012100
	HER2/neu Peptides	NCT02636582, NCT00194714, NCT04144023, NCT04024800, NCT04197687, NCT03384914
	Novel Peptides	NCT02826434, NCT03362060
	Personalised Peptide Vaccine Combination Therapy	NCT03606967, NCT02427581
	ESR1 Peptide	NCT04270149
Cervical & Ovarian Cancers	HPV E6/E7 Liposomes Combination Therapy	NCT04580771
	WT1 Peptides	NCT02737787
Colorectal Cancer	MUC1 Peptides	NCT02134925
Gastric Cancer	Multiple Peptides Combination Therapy	NCT03784040
Glioblastoma	CMV Peptide targets	NCT02864368
	Novel Peptides	NCT04116658
	Personalised Peptide Vaccine	NCT03223103
	Survivin Peptides	NCT02455557
	Telomerase-derived Peptides	NCT04280848
Glioma	Neoantigen Peptides	NCT04749641, NCT02358187, NCT01130077
	Neoantigen Combination Therapy	NCT03893903, NCT02960230
	Multiple Peptides Combination Therapy	NCT02924038
Head & Neck Cancers	IDO Peptides	NCT04445064
Kidney Cancer	Personalised Peptide Vaccine Combination Therapy	NCT02950766
Liver Cancer	PKA Peptide Combination Therapy	NCT04248569
Lung Cancer (inc. NSCLC)	MUC1 Peptides	NCT03300817, NCT01720836
	P10s-PADRE Peptide	NCT02264236
	Telomerase-derived Peptides	NCT01789099, NCT02818426
	Neoantigen Peptides	NCT04487093
	Personalised Peptide Vaccine	NCT04397926
Lymphoma	Novel Peptides	NCT04669171
	Personalised Peptide Vaccine Combination Therapy	NCT03361852
Melanoma	BRAF/CD4 Epitopes	NCT04364230
	CD4+ Epitope peptides	NCT03617328
	Novel Peptides	NCT02126579
	NY-ESO & gp100 Peptide Combination Therapy	NCT01176474, NCT01176461
	IDO & PD-L1 Peptide Combination Therapy	NCT03047928
	Personalised Peptide Vaccine Combination Therapy	NCT04072900
Multiple Cancers & Solid Tumours	Arginase-1 Peptide	NCT03689192
	HER2/neu Peptides	NCT01376505
	KRAS Peptide Combination Therapy	NCT04117087
	Multiple Peptides	NCT04316689
	Personalised Peptide Vaccine	NCT03715985
	Personalised Peptide Vaccine Combination Therapy	NCT03633110, NCT04266730
	Survivin Peptides Combination Therapy	NCT03879694
	Telomerase-derived Peptides Combination Therapy	NCT03946358
Myeloma	Novel Peptide Combination Therapy	NCT02886065
	PD-L1 Peptides	NCT03850522
Pancreatic Cancer	Neoantigen Peptides	NCT03956056
	Personalised Peptide Vaccine	NCT03558945
Prostate Cancer	Bcl-xl Peptides	NCT03412786
	Novel Peptide-Conjugate	NCT04701021
	RhoC Peptide	NCT04114825
	TARP Peptide	NCT02362464
	Telomerase-derived Peptides	NCT01784913

For peptide-based cancer vaccines to make their mark on cancer treatment, future studies will need to ensure a robust combination of *in vivo* CD4^+^ and CD8^+^ responses in a package that strongly activates DCs and subsequently T cells in a prolonged fashion, with minimal exhaustion or immune tolerance. They will need to be targeted, multi-faceted and personalised to an individual’s neoantigen repertoire, and able to overcome or reduce the immunosuppressive burden of the tumour microenvironment.

## Author Contributions

AS conceptualised and wrote the first draft of the manuscript. NB-B and SJ supervised. All authors contributed to the article and approved the submitted version.

## Funding

This research was funded by Innovate UK, grant number 104992 & 133783, and by grants from the CBI and Oxford Vacmedix UK Ltd.

## Conflict of Interest

SJ receives grants from CBI and Oxford Vacmedix UK Ltd, a spin-out from University of Oxford who develop recombinant overlapping peptide (ROP) cancer vaccines. NB-B consults for Oxford Vacmedix UK Ltd.
